# The prognostic value of absolute lymphocyte count and neutrophil‐to‐lymphocyte ratio for patients with metastatic breast cancer: a systematic review and meta‐analysis

**DOI:** 10.3389/fonc.2024.1360975

**Published:** 2024-03-07

**Authors:** Bulin Sang, Yuxin Fan, Xurao Wang, Lixian Dong, Yuanyuan Gong, Wenhong Zou, Guanhua Zhao, Jianchang He

**Affiliations:** ^1^ Clinical Pharmacology Research Center, Yunnan Provincial Hospital of Traditional Chinese Medicine, Kunming, China; ^2^ College of Pharmacy, Dali University, Dali, China; ^3^ Department of Clinical Pharmacy, 920th Hospital of Joint Logistics Support Force, Kunming, China

**Keywords:** ALC, NLR, MBC, prognostic marker, meta-analysis

## Abstract

**Background:**

Neutrophil‐to‐lymphocyte ratio (NLR) is considered a potential prognostic marker in early breast cancer. However, the prognosis of absolute lymphocyte count (ALC) and NLR in metastatic breast cancer (MBC) has been reported in a few studies, and conclusions are still conflicting. This present manuscript aims to provide further solid evidence regarding the prognostic values of ALC and NLR in MBC patients.

**Method:**

Eligible studies that reported the associations between ALC or NLR and MBC were included by searching relative electronic databases. Overall survival (OS) and progression-free survival (PFS) were used as outcome measures. The hazard ratio (HR) values and 95% confidence interval (CI) of the outcome measures were collected as effect sizes, and further analysis and discussion were conducted according to the pooled HR, subgroup analysis, publication bias, and interstudy heterogeneity.

**Results:**

Twenty-nine studies comprising 3,973 patients with MBC were included. According to our findings, lower ALC was significantly associated with poorer prognosis of OS (HR = 0.57, 95% CI 0.48 to 0.68) and PFS (HR = 0.68, 95% CI 0.58 to 0.79), and greater NLR was associated with poorer OS (HR = 1.50, 95% CI 1.35 to 1.67) and PFS (HR = 1.82, 95% CI 1.42 to 2.35). Furthermore, the prognostic values of ALC and NLR in MBC were also observed in the subgroup analyses regarding cutoff values and ethnicities.

**Conclusion:**

Low ALC and elevated NLR were observed to be significantly associated with adverse OS and PFS in MBC, indicating that ALC and NLR may act as potential prognostic biomarkers of MBC patients. Meanwhile, our results will also provide some novel evidence and research clues for the selection and development of clinical treatment strategies for MBC patients.

**Systematic review registration:**

https://www.crd.york.ac.uk/PROSPERO/, identifier CRD42021224114.

## 1 Background

Breast cancer has become the leading cause of morbidity and mortality in women worldwide (1). Most patients with early-stage breast cancer have a good prognosis, but metastatic breast cancer (MBC) is generally regarded as an incurable disease (2, 3). At present, distant metastasis and multiorgan metastasis remain a great challenge for disease recurrence and long-term survival in patients with MBC (4). It was estimated that the median overall survival (OS) of MBC patients was approximately 3 years, with a 5-year survival rate of approximately 25%, and patients with lung metastasis and bone metastasis were even less than 20% (4, 5). However, the underlying mechanisms of distant metastasis and colonization of breast cancer have not been elucidated (6). Furthermore, although multiple modalities or drug options are available in clinics, such as primary surgery and CDK4/6 inhibitors in combination with endocrine and dual anti-inhibitors, the prognosis of these patients is still unsatisfactory (7–10). Therefore, exploring appropriate prognostic biomarkers is of great clinical significance for improving prognosis and monitoring treatment and their response.

As a heterogeneous disease, the occurrence and development of MBC are closely related to inflammatory factors (11). In recent years, it has been found that absolute lymphocyte count (ALC) and neutrophil and platelet count may affect the progression of MBC (12). Pretreatment neutrophil-to-lymphocyte ratio (NLR) and platelet-to-lymphocyte ratio (PLR) and other immune and inflammatory biomarkers have been reported to be independent predictors of breast cancer prognosis (13–15). It was observed that these easy-to-obtain, non-invasive, and individualized peripheral blood biomarkers are of great prognostic value in numerous tumors including breast cancer, and increased NLR and PLR and decreased ALC may be associated with poor prognosis (16, 17). However, several previous meta-analyses only focus on breast cancer, and thus, there is still great uncertainty for the values of these potential prognostic biomarkers in MBC (2). Furthermore, although the ALC and NLR have been studied as prognostic markers of MBC in recent years, their prognostic values have not been determined uniformly, and different viewpoints still exist (18–20). Nevertheless, to the best of our knowledge, there is still a lack of relevant meta-analysis to provide relatively systematic and solid evidence regarding the prognostic value of ALC and NLR in MBC.

ALC and NLR, as major peripheral blood biomarkers that influence tumorigenesis and development, are expected to play a key role in the selection of drug therapies for patients with MBC in the future. Therefore, this study conducted a comprehensive and detailed meta-analysis of the effects of ALC and NLR on OS and progression-free survival (PFS) in patients with MBC and a subgroup discussion of factors affecting the assessment of their prognostic values, hoping to provide a useful reference for the long-term survival and improvement of the quality of life of patients.

## 2 Method

### 2.1 Search strategies

The Preferred Reporting Items for Systematic Reviews and Meta-Analyses (PRISMA) statement was strictly followed, and our study protocol was registered at the PROSPERO website (https://www.crd.york.ac.uk/PROSPERO/) before the start of the study. The identifier of the systematic review registration was PROSPERO CRD42021224114. Based on the prognostic correlation of ALC and NLR with MBC, the following keywords were formulated as search terms: “Metastatic breast cancer,” “Metastatic breast Neoplasms,” “Absolute lymphocyte count,” “Baseline lymphocyte count,” “ALC,” “neutrophil-to-lymphocyte ratio,” and “NLR.” The detailed search strategies are provided in **Supplementary Table 1**. A comprehensive literature search of relevant English language studies published up to April 2023 was conducted through online medical databases such as PubMed, EMBASE, the Cochrane Library, and Web of Science. In order to avoid omission of literature that met the inclusion criteria, the literature searches and backward searches were conducted independently based on the same strategy by the two researchers to collect eligible literature as detailed and complete as possible.

### 2.2 Inclusion and exclusion criteria

According to the pre-established inclusion and exclusion criteria, the title, abstract, and full-text article were screened. No restrictions were placed on the enrolled studies, including region, race, MBC subtype, age, and treatment regimen. When the results differed between the two researchers, a discussion with a third researcher was conducted and a final decision was made.

The inclusion criteria for the articles were as follows: 1) the study subjects are MBC patients; 2) the study evaluates the prognostic value of ALC or NLR in patients with MBC; 3) the study reports the results of the OS or PFS or provides available data to calculate the hazard ratio (HR) and 95% confidence interval (CI) of the OS or PFS; and 4) when the data are published repeatedly, the most recent study with the most detailed information will be selected.

The exclusion criteria for the articles were as follows: 1) reviews, case reports, comments, and conference abstracts are excluded; 2) studies about animals or cells are also excluded; and 3) studies with incomplete data are not considered.

### 2.3 Data extraction

After the two researchers independently completed the literature selection based on pre-designed criteria, the Excel and EndNote software were used to manage and extract the needed information. The OS and PFS, the primary survival outcomes, were extracted in the form of HR and 95% CI. Other detailed information was also extracted as follows: name of the first author, year of publication, country, study type, age of the patients, treatment, follow-up period, tumor subtype, number of metastatic sites, and cutoff values of ALC and NLR. If more than two or more sample sets appeared in the same study, the more complete and detailed data would been adopted. Further analysis is carried out when all researchers confirm the correctness of the data.

### 2.4 Quality assessment

The qualities of all the included studies were objectively assessed by two researchers using the standard Newcastle–Ottawa scale (NOS), which is composed of the following three quality indicators: outcome assessment, comparability, and selection. Each individual study was scored from 0 to 9 based on these parameters. The higher the score, the better the quality of the literature (21).

### 2.5 Statistical analysis

The pooled HR values were statistically analyzed using the Cochrane Collaboration’s Review Manager (version 5.3) and visually presented by forest plots. When the HRs and 95% CIs cannot be extracted from the table in the articles, these were indirectly acquired using the Engauge Digitizer (version 4.1) from the Kaplan–Meier graph (22). The random-effects model was used to evaluate the prognostic value of ALC and NLR in MBC patients with remarkable heterogeneity, and the fixed-effects model was adopted. Heterogeneity between studies was calculated through the Cochran *Q* and *I* ^2^ statistics, and the source of heterogeneity was estimated by subgroup analysis according to region, sample size, cutoff values, tumor subtype, and therapeutic strategies. At the same time, publication bias and sensitivity analysis of the main outcomes were also performed when applicable by using the Stata software (version 15.0) (23).

## 3 Results

### 3.1 Characteristics of the included studies

**Figure 1:**
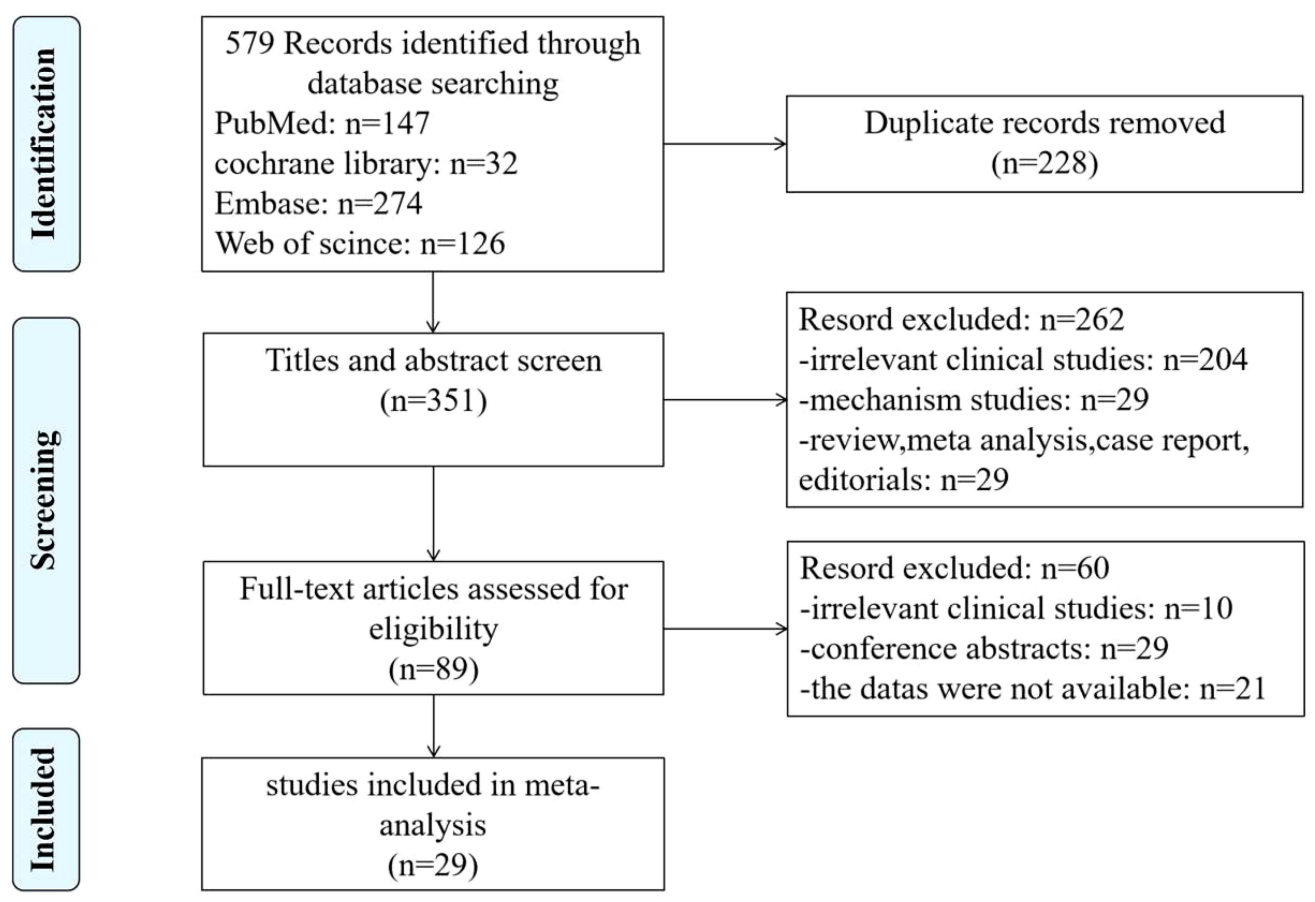
Selection process of the studies included.

A total of 579 articles were retrieved from four databases, consisting of 147 from PubMed, 32 from the Cochrane Library, 274 from EMBASE, and 126 from the Web of Science. After reviewing all the literature, 351 articles were included through screening of the title, abstract, and full text. Finally, 29 articles were included in this meta‐analysis after strict screening (18–20, 24–49) (**Figure 1**). The information of all the included literature was collected and pooled, and the basic characteristics are described in **Table 1**. Twenty-nine studies were from nine countries, 28 of which were published within the last 5 years, and the average age of the included 3,952 patients was 51–63 years. Most of the patients have visceral metastasis and two-organ metastasis. Only 14 studies analyzed the prognostic value of ALC and 26 studies discussed the effects of NLR on MBC patients. The primary cutoff values of 1,500 and 3 were used in most of the studies. Detailed information is shown in **Table 2**. All NOS scores of the included studies were 8–9, suggesting high quality according to the quality assessment.

**Table 1:** Characteristics of the included studies.

Study	Year	Country	Study type	Sample size	Ages (years)	Treatment	Endpoint	NOS scores
Sawa	2022	Japan	Single center	243	58 (22–90)	Hormonal therapy Chemotherapy	OS	9
Jimbo	2022	Japan	Single center	108	56 (32–86)	Endocrine therapy Chemotherapy	OS	8
Emile	2022	France	Single center	114	51 (30–75)	CDK4/6 inhibitor plus Endocrine therapy	OS and PFS	8
Inoue	2022	Japan	Single center	131	NR	Chemotherapy Endocrine therapy Anti-HER2 therapy	OS	9
Shikanai	2022	Japan	Single center	107	58 (35–87)	CDK4/6 inhibitor CDK4/6 inhibitor plus Endocrine therapy	PFS	8
Xiang	2022	China	Single center	94	51 (46–62)	Chemotherapy	OS	9
Takamizawa	2022	Japan	Single center	91	NR	Eribulin Capecitabine	OS and PFS	9
Shao	2022	China	Single center	129	51 (25–82)	Anti-HER2 therapy T-DM1 TKI	OS and PFS	9
Yilmaz	2022	Turkey	Single center	101	56 (26–88)	CDK4/6 inhibitor plus Endocrine therapy	OS and PFS	8
Koyama	2021	Japan	Single center	120	61 (20–82)	Eribulin Adjuvant or Neoadjuvant Chemotherapy	OS and PFS	8
Nakamoto	2021	Japan	Single center	94	62 (37–83)	Eribulin	OS	8
Morisaki	2021	Japan	Single center	88	NR	Eribulin	OS and PFS	8
Oba	2021	Japan	Single center	60	58.6 ± 11.9	Eribulin	OS and PFS	8
Miyagawa	2020	Japan	Multicenter	179	NR	Bevacizumab plus Paclitaxel	OS and PFS	8
Gerratana	2020	Italy	Single center	396	NR	NR	OS	9
Sata	2020	Japan	Single center	74	NR	Eribulin Adjuvant or Neoadjuvant Chemotherapy	OS and PFS	7
Myojin	2020	Japan	Single center	104	59 (38–82)	Eribulin Anti-HER2 therapy	PFS	8
de la Cruz-Ku	2020	Peru	Single center	118	NR	Chemotherapy Others	OS	9
Liu	2020	China	Single center	176	56	Chemotherapy Endocrine therapy Targeted therapy Others	OS and PFS	9
Ueno	2020	Japan	Single center	125	57	Eribulin	OS and PFS	8
De Giorgi	2019	USA	Single center	280	NR	Systemic treatment	OS	8
Che	2019	China	Single center	68	51 (27–74)	Anti-HER2 therapy Chemotherapy	OS and PFS	9
Ivars Rubio	2019	Spain	Single center	263	59 (19–95)	Chemotherapy Endocrine therapy Chemotherapy plus Biological agents Anti-HER2 therapy Anti-VEGF therapy	OS and PFS	9
Imamura	2019	Japan	Multicenter	53	NR	T-DM1	OS and PFS	8
Blanchette	2018	Canada	Single center	154	56 (47–63)	Anti-HER2 therapy	OS	8
Takuwa	2018	Japan	Single center	171	59 (31–92)	Multidisciplinary therapy	OS	9
Miyagawa	2018	Japan	Single center	59	63 (34–83)	Eribulin Others	OS and PFS	8
Vernieri	2018	Italy	Single center	57	56 (33.7–78.9)	Chemotherapy	OS and PFS	8
De Giorgi	2012	USA	Single center	195	54 (24–84)	Systemic treatment	OS and PFS	8
Models	Cutoff	Follow‐up (months)	Tumor subtype (100%)	Visceral metastasis (%)	Number of metastatic sites (%)			
ALC and NLR	ALC: 1,500; NLR: 3	26.4 (0.1–192.1)	NR	159 (65.4)	NR			
ALC and NLR	ALC: 1,629; NLR: 1.99	NR	NR	NR	NR			
ALC	ALC: 1,500	NR	HR^+^/HER2^−^	NR	NR			
NLR	NLR: 2.52	59 (6–151)	NR	NR	≥2; 12 (9.2)			
ALC and NLR	ALC:1,505; NLR: 2.5	NR	ER^+^/HER2^−^	67 (62.6)	NR			
NLR	NLR: 2.285	30 (2–109)	NR	NR	NR			
ALC and NLR	ALC: 1,500; NLR: 3	19.1	NR	NR	NR			
NLR	NLR: 3	21.0 (2.0–46.0)	HER2^+^	89 (69.0)	>2; 41 (31.8)			
NLR	NLR: 2.19	NR	HR^+^/HER2^−^	32 (31.7)	≥2; 49 (48.5)			
ALC and NLR	ALC: 1,285; NLR: 3.3	15.2 (0.9–65.9)	HER2^−^	100 (83.3)	≥2; 58 (48.3)			
ALC and NLR	ALC: 1,500; NLR: 3	NR	HER2^−^	77 (81.9)	≥3; 75 (79.8)			
ALC	ALC: 1,500	15.9 (1.7–75.6)	NR	64 (72.7)	NR			
NLR	NLR: 2.32	13.7 (1.63–54.17)	NR	48 (80.0)	NR			
ALC and NLR	ALC: 1,500; NLR: 3	NR	NR	144 (80.4)	NR			
NLR	NLR: 2	53	NR	NR	≥2; 183 (46.2)			
ALC and NLR	ALC: 1,500; NLR: 3	NR	NR	34 (45.9)	NR			
ALC and NLR	ALC: 1236; NLR: 3.3	NR	NR	75 (72.1)	NR			
NLR	NLR: 2.5	24	Triple negative	NR	≥2; 60 (50.8)			
NLR	NLR: 2.085	25.4	NR	77 (43.8)	NR			
ALC and NLR	ALC: 1,500; NLR: 5	NR	NR	91 (72.8)	NR			
NLR	NLR: 3	NR	NR	NR	NR			
ALC and NLR	ALC: 1,000; NLR: 3	26.5 (2.28–97.2)	HER2^+^	NR	≥2; 44 (64.7)			
NLR	NLR: 2.32	44.9 (6–107)	NR	65 (24.7)	≥2; 111 (42.2)			
NLR	NLR: 2.56	NR	HER2^+^	37 (69.8)	≥3; 26 (49.1)			
NLR	NLR: 3.18	NR	HER2^+^	NR	≥2; 70 (45.8)			
NLR	NLR: 1.9	44 (0–271)	NR	120 (70.2)	≥2; 119 (69.6)			
NLR	NLR: 3	NR	NR	44 (74.6)	NR			
NLR	NLR: 2.5	NR	Triple negative	37 (64.9)	>2; 21 (36.8)			
ALC	ALC: 1,000	NR	NR	NR	NR			

OS, overall survival; PFS, progression-free survival; NOS, Newcastle–Ottawa scale; NR, not reported; ALC, absolute lymphocyte count; NLR, neutrophil to lymphocyte ratio; T-DM1, trastuzumab emtansine; TKI, tyrosine kinase inhibitor.

**Table 2:** Subgroup analyses of ALC for OS and PFS.

Subgroups	Independent cohorts	HR (95% CI) (H/L*)	*p*-value	Heterogeneity	
				*I* ^2^, %	*p*-value
**Overall survival**	**12**				
Cutoff value					
>1,500	1	0.13 [0.02, 0.72]	0.02	–	–
1,500	8	0.59 [0.48, 0.73]	<0.00001	0	0.56
<1,500	3	0.56 [0.42, 0.76]	0.0002	49	0.14
Region					
Asia	10	0.60 [0.49, 0.72]	<0.00001	23	0.23
America	1	0.45 [0.27, 0.73]	0.001	–	–
Europe	1	0.57 [0.33, 0.97]	0.04	–	–
Sample size					
≥100	7	0.59 [0.49, 0.72]	<0.00001	25	0.24
<100	5	0.49 [0.33, 0.72]	0.0003	0	0.41
Treatment					
Eribulin	4	0.64 [0.44, 0.93]	0.15	43	0.02
Chemotherapy	1	0.57 [0.28, 1.18]	0.13	–	–
CDK4/6 inhibitor plus Endocrine therapy	1	0.57 [0.33, 0.97]	0.04	–	–
Bevacizumab plus Paclitaxel	1	0.53 [0.35, 0.80]	0.003	–	–
Tumor subtype					
HER2^+^	1	0.33 [0.13, 0.81]	0.02	–	–
HER2^−^	2	0.51 [0.20, 1.26]	0.06	72	0.14
HR^+^/HER2^−^	1	0.57 [0.33, 0.97]	0.04	–	–
**Progression-free survival**	**10**				
Cutoff value					
**>**1,500	1	0.75 [0.29, 1.93]	0.55	–	–
1,500	5	0.65 [0.52, 0.82]	0.0003	0	0.67
<1,500	4	0.69 [0.56, 0.86]	0.0007	76	0.006
Region					
Asia	8	0.74 [0.61, 0.89]	0.001	44	0.08
America	1	0.55 [0.38, 0.79]	0.001	–	–
Europe	1	0.59 [0.38, 0.89]	0.01	–	–
Sample size					
≥100	7	0.70 [0.59, 0.82]	<0.00001	18	0.3
<100	3	0.59 [0.41, 0.85]	0.005	72	0.03
Treatment					
Eribulin	2	0.73 [0.49, 1.08]	0.2	39	0.12
Chemotherapy	1	0.68 [0.38, 1.22]	0.19	–	–
CDK4/6 inhibitor plus Endocrine therapy	1	0.59 [0.38, 0.89]	0.01	–	–
Bevacizumab plus Paclitaxel	1	0.69 [0.43, 1.11]	0.12	–	–
Tumor subtype					
HER2^+^	1	0.26 [0.12, 0.55]	0.0005	–	–
HER2^−^	1	1.02 [0.71, 1.48]	0.91	–	–
HR^+^/HER2^−^	1	0.59 [0.38, 0.89]	0.01	–	–
ER^+^/HER2^−^	1	0.75 [0.29, 1.93]	0.55	–	–

No statistical results were in line.

HR, hazard ratio; CI, confidence interval; H, high; L, low.

### 3.2 Correlation between ALC and OS in MBC patients

**Figure 2:**
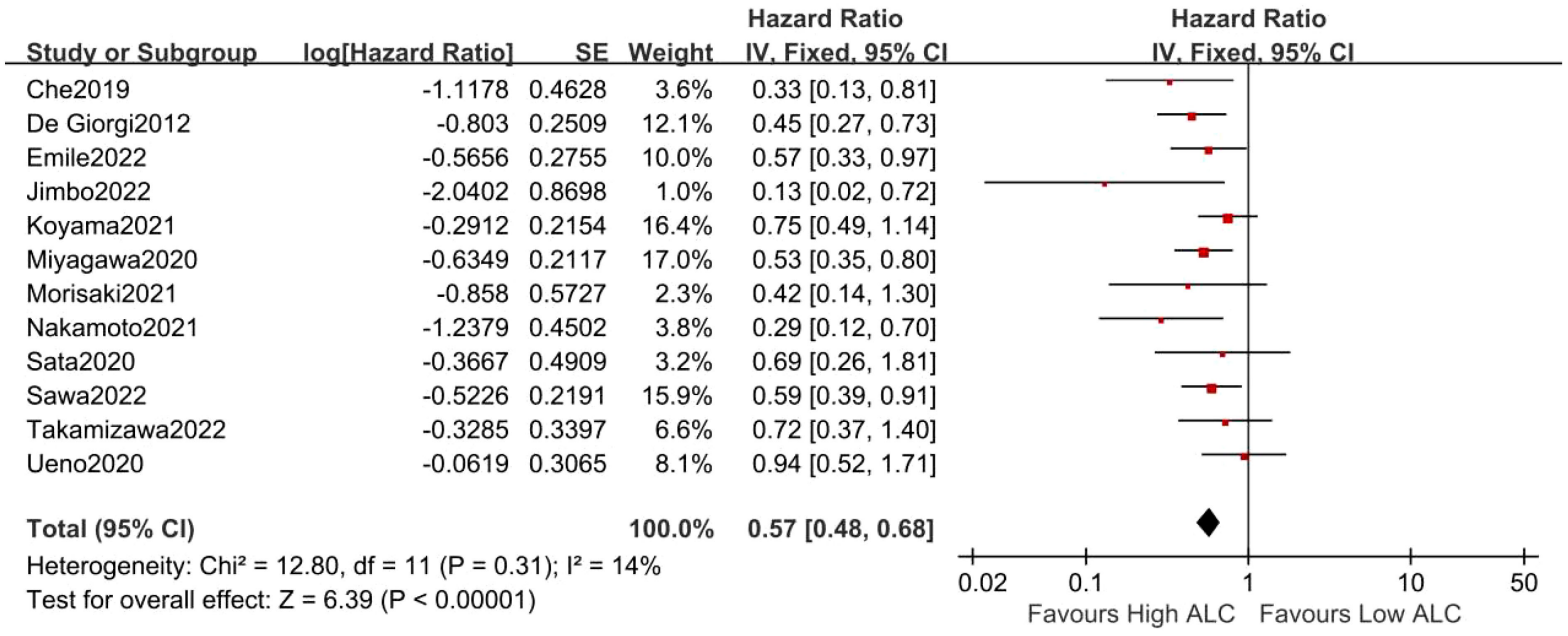
Forest plot of ALC for OS.

A total of 1,584 MBC patients from 12 studies were enrolled in this present meta-analysis to assess the correlation between ALC and OS. The pooled analysis of all studies demonstrated that low ALC was significantly associated with poor OS (fixed-effects model, HR = 0.57, 95% CI = 0.48 to 0.68, *p* < 0.01) with low heterogeneity (*I* ^2^ = 14%, *p* = 0.31, **Figure 2**).

### 3.3 Correlation between ALC and PFS in MBC patients

**Figure 3:**
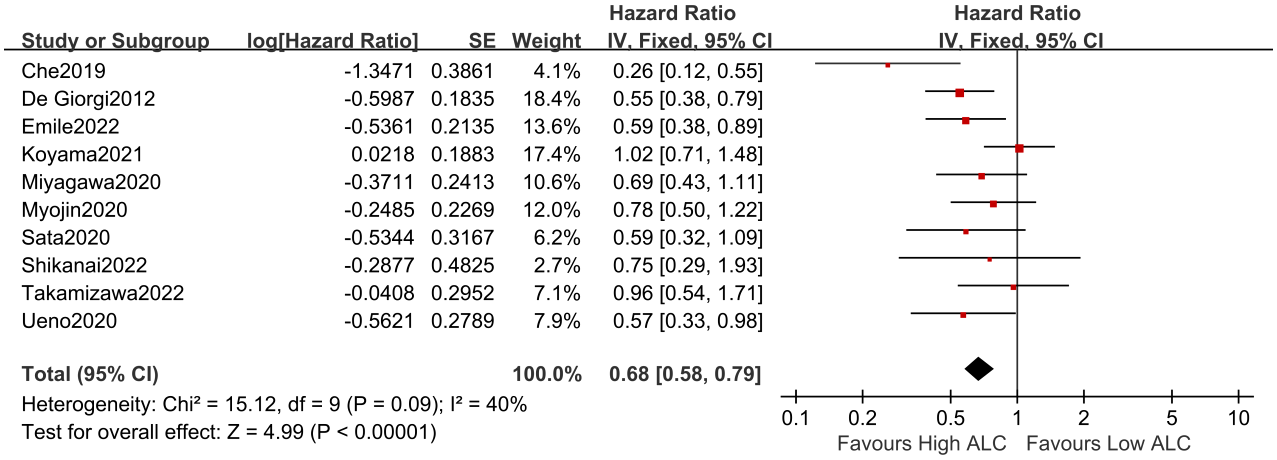
Forest plot of ALC for PFS.

A total of 10 studies involving 1,262 MBC patients were incorporated to estimate the connection between ALC and PFS. The combined analysis of all the studies indicated that a lower ALC level was related to a shorter PFS (fixed-effects model, HR = 0.68, 95% CI = 0.58 to 0.79, *p* < 0.01) with low heterogeneity (*I* ^2^ = 40%, *p* = 0.09, **Figure 3**).

#### 3.4 Correlation between NLR and OS in MBC patients

**Figure 4:**
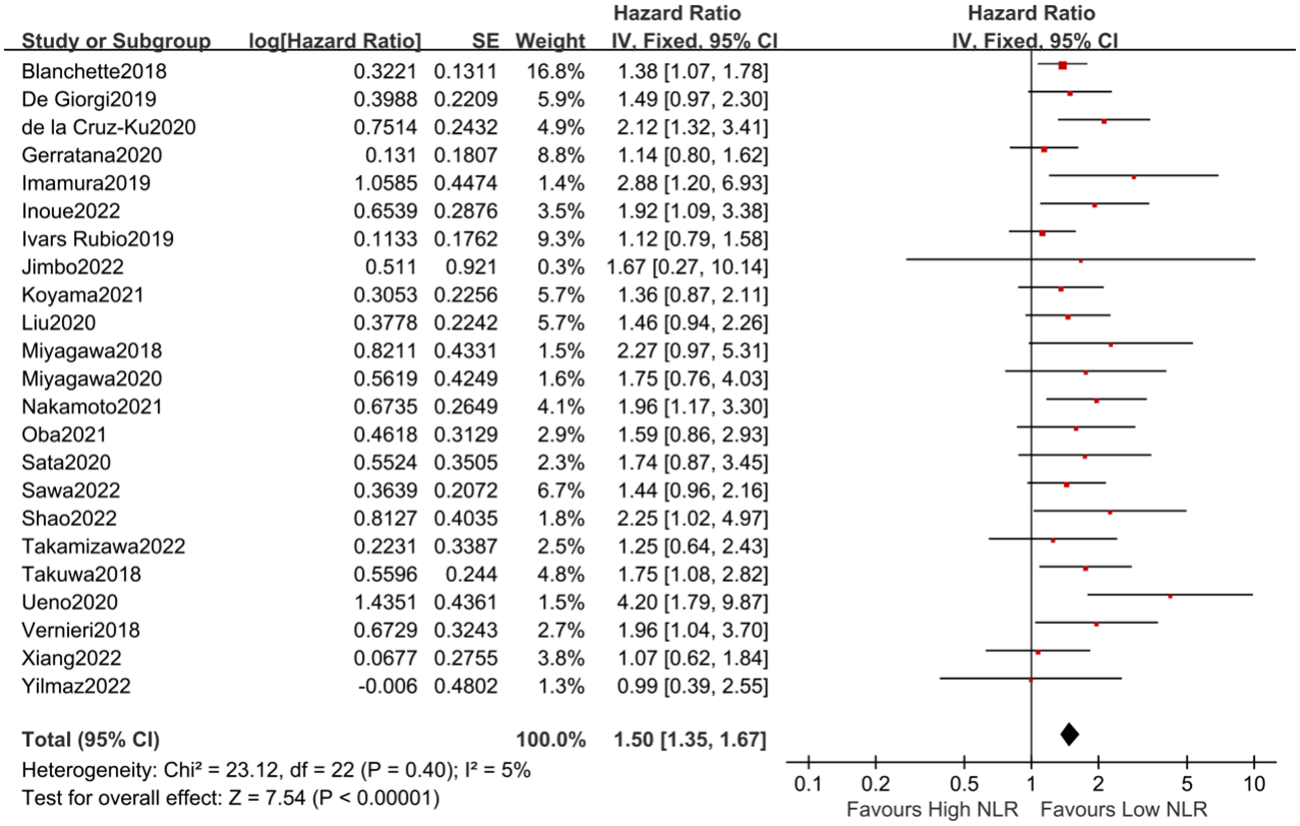
Forest plot of NLR for OS.

Twenty-three studies with 3,276 MBC patients appraised the association between NLR and OS. Nine studies showed that NLR may be a potential prognostic biomarker for OS, but no remarkable correlation between NLR and OS was observed in 14 publications. However, according to the pooled analysis, it was demonstrated that high NLR values were obviously associated with poor OS (fixed-effects model, HR = 1.50, 95% CI = 1.35 to 1.67, *p* < 0.01) with low heterogeneity (*I* ^2^ = 5%, *p* = 0.40, **Figure 4**).

### 3.5 Correlation between NLR and PFS in MBC patients

**Figure 5:**
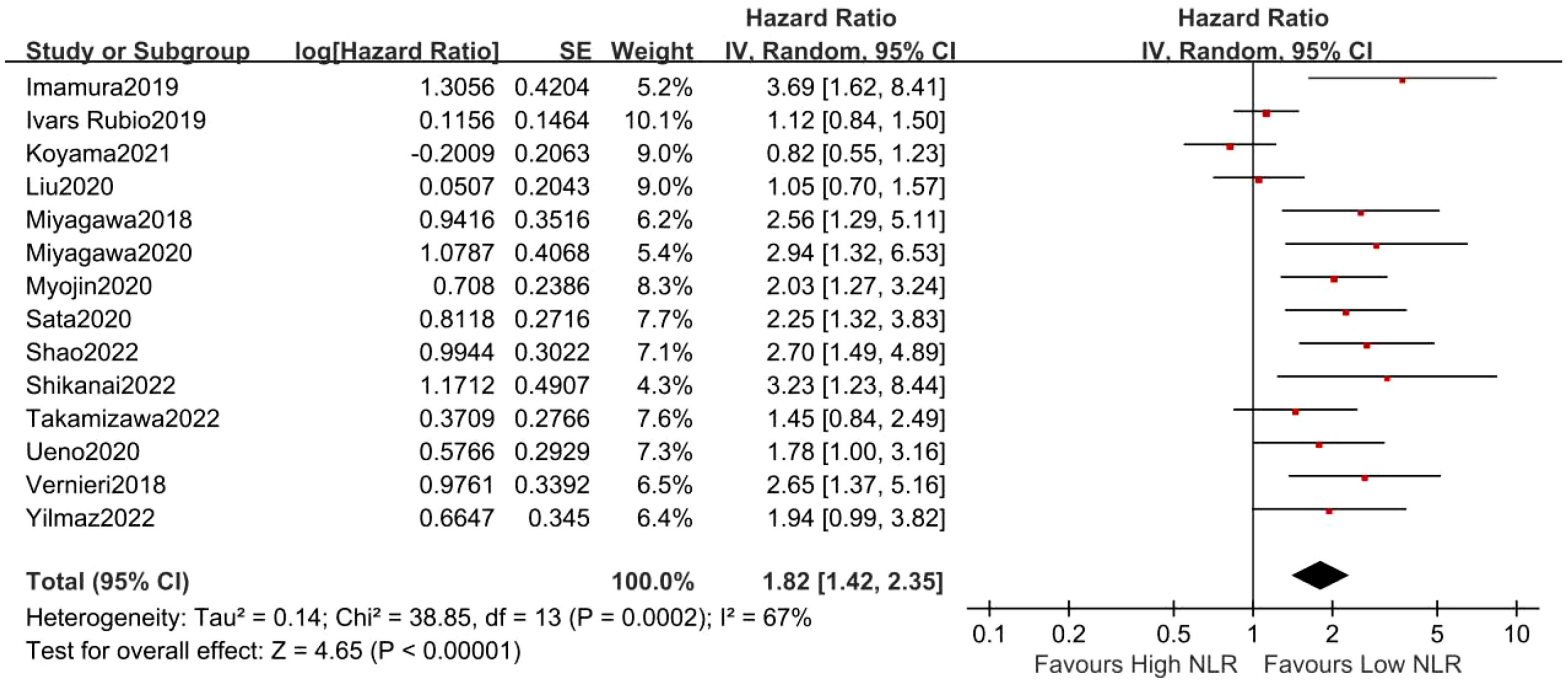
Forest plot of NLR for PFS.

Fourteen studies with 1638 MBC patients evaluated the relationship between the NLR and PFS. The pooled outcome showed that higher NLR was markedly connected with adverse PFS (random-effects model, HR = 1.82, 95% CI = 1.42 to 2.35, *p* < 0.01) with high heterogeneity (*I* ^2^ = 80%, *p* = 0.0002, **Figure 5**).

### 3.6 Subgroup analyses for the relationship between ALC and OS/PFS

Based on the extracted data and influencing factors, subgroup analysis was performed on the study region, cutoff value, sample size, treatment, and tumor subtype. The effects of ALC on the OS and PFS of patients with MBC among different subgroups are shown in **Table 2**.

It was found that the significant relationship between ALC and OS was not affected by the cutoff value, region, and sample size, suggesting that ALC might be a promising biomarker for OS in MBC patients. Moreover, the high ALC was significantly associated with better OS in MBC patients given CDK4/6 inhibitor plus endocrine therapy (HR = 0.57, 95% CI = 0.33 to 0.97, *p* = 0.04) and bevacizumab plus paclitaxel therapy (HR = 0.53, 95% CI = 0.35 to 0.80, *p* = 0.003), and the association between ALC and OS was also observed in MBC patients with HER2^+^ (*p* = 0.02) and HR^+^/HER2^−^ (*p* = 0.04), but since only one study was involved, further high-quality research with a large sample is required in the future.

As for the subgroup analysis of the relationship between ALC and PFS, although no relationship between ALC and PFS was observed in one study with an ALC cutoff value of >1,500, the remaining results of the ALC cutoff value of ≤1,500, region, and sample size further indicated that high ALC was associated with better PFS. Likewise, the subgroup analysis regarding treatment and tumor subtype involved extremely limited studies, and therefore, a larger number of studies with bigger sample sizes need to be further explored.

### 3.7 Subgroup analyses for the relationship between NLR and OS/PFS

As shown in **Table 3**, the subgroup analyses based on the cutoff value of NLR and sample sizes also demonstrated the promising prognostic value of NLR in OS. Furthermore, it was observed that higher NLR was associated with worse PFS in patients treated with bevacizumab plus paclitaxel (HR = 2.03, 95% CI = 1.27 to 3.24, *p* = 0.003) and T-DMI (HR = 3.69, 95% CI = 1.62 to 8.41, *p* = 0.002). Additionally, similar results were found in MBC patients with ER^+^/HER2^−^ (HR = 3.23, 95% CI = 1.23 to 8.44, *p* = 0.02) and triple-negative breast cancer (HR = 2.65, 95% CI = 1.37 to 5.16, *p* = 0.004). However, some non-significant results were presented in certain subgroup analyses on treatment and tumor subtype, which may account for the limited number of studies, and further solid evidence is required based on high-quality studies with large samples.

**Table 3:** Subgroup analyses of NLR for OS and PFS.

Subgroups	Independent cohorts	HR (95% CI) (H/L*)	*p*-value	Heterogeneity	
				*I* ^2^, %	*p*-value
**Overall survival**	**23**				
Cutoff value					
>3	3	1.47 [1.19, 1.83]	0.0004	67	0.05
3	8	1.63 [1.33, 2.01]	<0.00001	0	0.89
<3	12	1.44 [1.24, 1.68]	<0.00001	16	0.29
Region					
Asia	17	1.62 [1.40, 1.88]	<0.00001	0	0.61
America	3	1.51 [1.24, 1.85]	<0.00001	17	0.3
Europe	3	1.21 [0.96, 1.53]	0.1	20	0.28
Sample size					
≥100	15	1.46 [1.29, 1.64]	<0.00001	13	0.3
<100	8	1.66 [1.32, 2.08]	<0.00001	0	0.54
Treatment					
Eribulin	4	1.86 [1.35, 2.55]	0.16	42	0.0001
Chemotherapy	3	1.17 [0.65, 2.12]	0.12	53	0.60
CDK4/6 inhibitor plus Endocrine therapy	1	0.99 [0.39, 2.55]	0.99	–	–
Bevacizumab plus Paclitaxel	1	1.75 [0.76, 4.03]	0.19	–	–
T-DMI	1	2.88 [1.20, 6.93]	0.02	–	–
Anti-HER2 therapy	1	1.38 [1.07, 1.78]	0.01	–	–
Tumor subtype					
HER2^+^	3	1.52 [1.20, 1.92]	0.17	44	0.0005
HER2^−^	2	1.58 [1.13, 2.22]	0.29	11	0.007
HR^+^/HER2^−^	1	0.99 [0.39, 2.55]	0.99	–	–
Triple negative	2	2.06 [1.41, 3.02]	0.85	0	0.0002
**Progression-free survival**	**14**				
Cutoff value					
>3	3	1.41 [0.77, 2.59]	0.27	79	0.008
3	5	2.20 [1.68, 2.90]	<0.00001	0	0.49
<3	6	1.81 [1.19, 2.77]	0.006	70	0.0005
Region					
Asia	12	1.88 [1.42, 2.50]	<0.00001	64	0.001
Europe	2	1.63 [0.71, 3.77]	0.25	82	0.02
Sample size					
≥100	9	1.61 [1.19, 2.20]	0.002	69	0.001
<100	5	2.24 [1.67, 3.00]	<0.00001	7	0.37
Treatment					
Eribulin	2	1.60 [1.08, 2.37]	0.61	0	0.02
Chemotherapy	2	1.79 [0.80, 4.03]	0.11	62	0.16
CDK4/6 inhibitor plus Endocrine therapy	1	1.94 [0.99, 3.82]	0.05	–	–
Bevacizumab plus Paclitaxel	1	2.03 [1.27, 3.24]	0.003	–	–
T-DMI	1	3.69 [1.62, 8.41]	0.002	–	–
Tumor subtype					
HER2^+^	2	3.01 [1.86, 4.86]	0.55	0	<0.00001
HER2^−^	1	0.82 [0.55, 1.23]	0.33	–	–
HR^+^/HER2^−^	1	1.94 [0.99, 3.82]	0.05	–	–
ER^+^/HER2^−^	1	3.23 [1.23, 8.44]	0.02	–	–
Triple negative	1	2.65 [1.37, 5.16]	0.004	–	–

No statistical results were in line.

HR, hazard ratio; CI, confidence interval; H, high; L, low; T-DMI, trastuzumab emtansine.

### 3.8 Sensitivity analysis and publication bias

**Figure 6:**
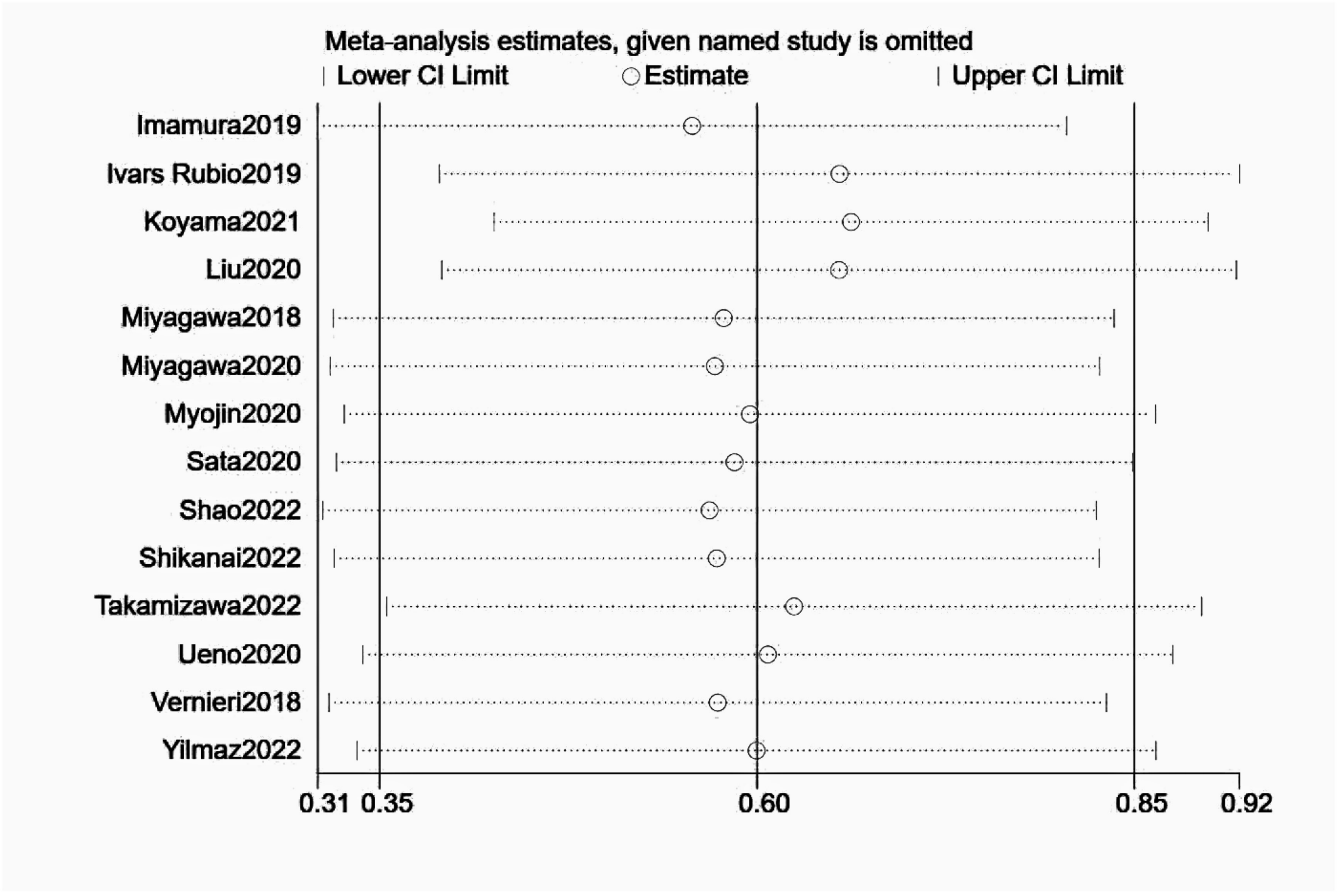
Sensitivity analyses of NLR for PFS.

Further sensitivity analysis was performed and presented in **Figure 6**. It was indicated that the pooled HRs and 95% CIs did not alter significantly, suggesting that these results were robust.

**Figure 7:**
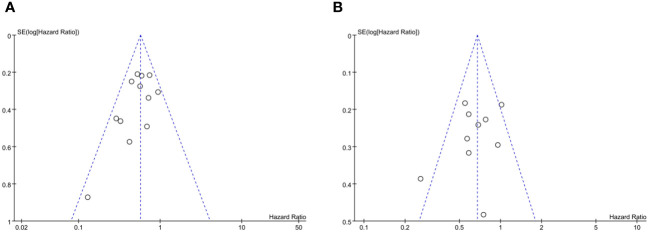
Funnel plot **(A)** and Egger’s test **(B)** of NLR for PFS.

The publication bias of the included studies was evaluated, and the corresponding results are presented in **Supplementary Figures 1, 2**. No potential publication bias was observed regarding the results of the association between ALC and OS/PFS (*p* > 0.05) and between NLR and OS (*p* > 0.05). However, as for the NLR and PFS, the funnel plot was obviously asymmetric and *p <*0.001, indicating that potential publication bias probably existed (**Figures 7A, B**).

## 4 Discussion

With the increasing incidence and mortality rate of breast cancer, more and more studies are devoted to investigating certain available and highly sensitive biomarkers of diagnosis and prognosis for early screening and prognosis monitoring of patients. If the simple and accessible, non-invasive biomarker in peripheral blood samples can be found to monitor the response to treatment based on the baseline level before treatment, the timely adjustment of the dose of treatment and the combination of drugs may be able to reduce the suffering and financial pressure of the patients. ALC and NLR were previously reported for different cancer types as possible prognostic indicators, but these were only retrospective data and were globally inconclusive according to their heterogeneity. Furthermore, it still remains uncertain as to what extent systemic inflammatory markers are directly involved in immune response, which may require further investigation by conducting additional studies. As the first systematic review and meta-analysis to investigate the prognostic value of ALC and NLR in MBC patients, we found that the low ALC and high NLR were significantly associated with poor prognosis of patients with MBC, particularly in Asian populations, suggesting that these two biochemical markers may act as promising biomarkers for prognosis in human MBC.

As a prognostic marker, NLR has attracted the attention of many researchers in the treatment of early breast cancer and other tumors. A systematic review and meta-analysis of the complete pathological response to neoadjuvant therapy in breast cancer investigated the prognostic value of NLR (50). Although the overall results showed that lower NLR was associated with higher complete pathological response, it did not reach statistical significance in a 5-year disease-free survival (DFS). Furthermore, Xue et al. also failed to confirm the prognostic value of NLR in DFS and OS after neoadjuvant therapy in breast cancer patients, which may be related to the reason that only three studies reporting OS and five studies reporting DFS were included, and the included studies were highly heterogeneous (51). Interestingly, in recent studies, Zhou et al. reported that high NLR was significantly associated with poor prognosis of OS and DFS in patients treated with neoadjuvant therapy (52). Therefore, whether high NLR can be used as a biomarker of poor prognosis on perioperative treatment for early-stage breast cancer still needs further study. Other studies conducted by Cupp et al. (53) and Templeton et al. (54) investigated the prognostic value of NLR in patients with early breast cancer and other cancers and also reached a similar discrepant conclusion. However, these previous studies did not perform detailed analysis on the relationship between NLR and complex MBC. The prognostic value of NLR on MBC was mentioned by Guo et al. (17) in a subgroup analysis, but only three relevant studies were included. Therefore, the prognostic effects of NLR in MBC still need to be further explored by studies with larger sample sizes and a high level of evidence. Thus, we conducted a systematic review and meta-analysis comprehensively studying the relationship between NLR and MBC.

In addition, more evidence indicated that the prognostic value of ALC is mainly presented in the treatment of inflammatory diseases (55–57). There are few studies on the prognostic value of ALC in cancer, most of which are single clinical studies. It was found that lymphopenia increased the risk of death in lung cancer patients treated with chemotherapy and immunosuppressive therapy, and low baseline lymphocyte count was a risk factor for poor survival (58). Feng et al. also reported similar results regarding the roles of ALC in diffuse large B-cell lymphoma (59). Although the types of cancer in the two studies differed from those in the present study, the conclusions concerning low ALC and poor prognosis were consistent. The relationship between ALC and the prognosis of MBC has received much attention and controversy in recent years. However, the prognostic role of ALC in MBC that can provide a reference for patients in clinical practice still needs to be verified. Fortunately, our present study supplies further evidence for this vacancy.

The conclusion that NLR and ALC are associated with the prognosis of MBC is mostly based on clinical evidence, and the mechanisms between them still remain unclear. Some studies believed that inflammatory processes play a significant role in supporting or inhibiting tumor progression and metastasis, and the changes in inflammatory cells are related to the occurrence and development of tumors (60). As an important indicator of inflammatory immunity, the number of neutrophils and lymphocytes can affect the proliferation, angiogenesis, and distant metastasis of tumor cells by secreting related cytokines and chemokines (54). IL-1β (61), IL-6 (62), IL-10 (63), etc. not only lead to epigenetic modifications (methylation of DNA) but also promote the activation of epithelial–mesenchymal transition (EMT), tumor cell homing, and positive feedback amplification of the protumorigenic inflammatory loop between tumor and resident cells (64). Just like this, the individual or mutual ratio of inflammatory immune cells such as neutrophils, lymphocytes, and monocytes has aroused the strong interest of researchers and is considered an important index to evaluate the prognosis of patients with inflammation or tumor (65). Previous studies have explored them as a poor prognostic factor affecting breast cancer patients, but the exact mechanism is still under study.

In our study, all of the included studies were of high quality, and the description of the statistical analysis and results was relatively cautious and objective. The advantages and disadvantages of other similar studies and the trend of current research were discussed objectively, and the conclusions were considered to be stable and reliable in agreement with the findings of this study. This is the first systematic review and meta-analysis to evaluate the relationship between ALC/NLR and the prognosis of MBC, which confirms the prognostic value of ALC/NLR in the treatment of MBC and provides further reference for the clinical research and clinical application of ALC and NLR in the future.

Admittedly, although the process of this systematic review and meta-analysis was strictly controlled, there were still some limitations. First, at the phase of raw data extraction, some studies only reported univariate hazard ratios, which may affect the pooled results of effect sizes and thus cause an overestimation of the conclusions. Second, the prognosis of patients with MBC may be affected by complex and multiple factors, such as cancer subtypes, cancer stages, stage of tumor progress, metastatic organs or numbers, detection methods, treatment options, and regions. The heterogeneous effects of these factors should be further studied when applicable. However, the lack of original data or the limited number of studies makes it impossible to perform subgroup analysis or obtain more valid and robust results based on the subgroup analyses. Third, all studies were comparable based on the baseline characteristics, but the included patients may have multisystem invasion and other underlying inflammatory diseases, which may also affect the level of ALC and NLR. Fourth, statistical heterogeneity was observed in the results on the prognostic value of NLR in PFS, so the clinical application of this part of the conclusion should be considered with more caution. Meanwhile, in future studies, investigators should fully take these limitations into consideration.

In conclusion, low ALC and high NLR were significantly associated with poor OS and PFS according to our results, indicating that ALC and NLR might be potential prognostic markers for patients with MBC. The application of these two simple and accessible, non-invasive and individualized prognostic indicators may provide a better reference on the choice of treatment for patients with MBC in the future. Meanwhile, some high-quality clinical studies with large samples are required to further validate these findings in the future.

## Data availability statement

The original contributions presented in the study are included in the article/**Supplementary Material**. Further inquiries can be directed to the corresponding author.

## Author contributions

BS: Writing – original draft, Writing – review & editing, Formal analysis, Methodology, Software, Supervision. YF: Formal analysis, Methodology, Software, Writing – original draft, Writing – review & editing. XW: Formal analysis, Methodology, Software, Writing – original draft, Writing – review & editing. LD: Data curation, Writing – original draft. YG: Data curation, Writing – original draft. WZ: Supervision, Writing – review & editing. GZ: Supervision, Writing – review & editing. JH: Funding acquisition, Supervision, Writing – review & editing.
