# Supplementary material for: The prognostic value of absolute lymphocyte count and neutrophil‐to‐lymphocyte ratio for patients with metastatic breast cancer: a systematic review and meta‐analysis
Source: Front Oncol. 2024 Mar 7;14:1360975. doi: 10.3389/fonc.2024.1360975 (PMC10955091; doi:10.3389/fonc.2024.1360975)
Supplement: Supplementary file 1 [file DataSheet_1.docx]

**Supplementary Table S1 Search strategies**

| **Search** | **Results** |
| --- | --- |
| 1. PubMed   #1. ((Metastatic breast cancer) OR (Metastatic breast Neoplasms)) OR (MBC)  #2. ((((Absolute lymphocyte count) OR (Baseline lymphocyte count)) OR (ALC)) OR (neutrophil to-lymphocyte ratio)) OR (NLR)  #3. #1 AND #2 | 147 |
| (B) Cochrane Library  #1. ((Metastatic breast cancer) OR (Metastatic breast Neoplasms)) OR (MBC)  #2. ((((Absolute lymphocyte count) OR (Baseline lymphocyte count)) OR (ALC)) OR (neutrophil to-lymphocyte ratio)) OR (NLR)  #3. #1 AND #2 | 32 |
| (C) Embase  1. Metastatic AND breast AND cancer OR (Metastatic AND breast AND Neoplasms) OR MBC  2. Absolute AND lymphocyte AND count OR (Baseline AND lymphocyte AND count) OR ALC OR (neutrophil AND to-lymphocyte AND ratio) OR NLR  3. 1 AND 2 | 274 |
| (D) web of science  1. ((TS=(Metastatic breast cancer) OR TS=(Metastatic breast Neoplasms)) OR TS=(MBC)  2. ((((TS=(Absolute lymphocyte count) OR TS=(Baseline lymphocyte count)) OR TS=(ALC)) OR TS=(neutrophil to-lymphocyte ratio)) OR TS=(NLR)  3. #1 AND #2 | 126 |

**
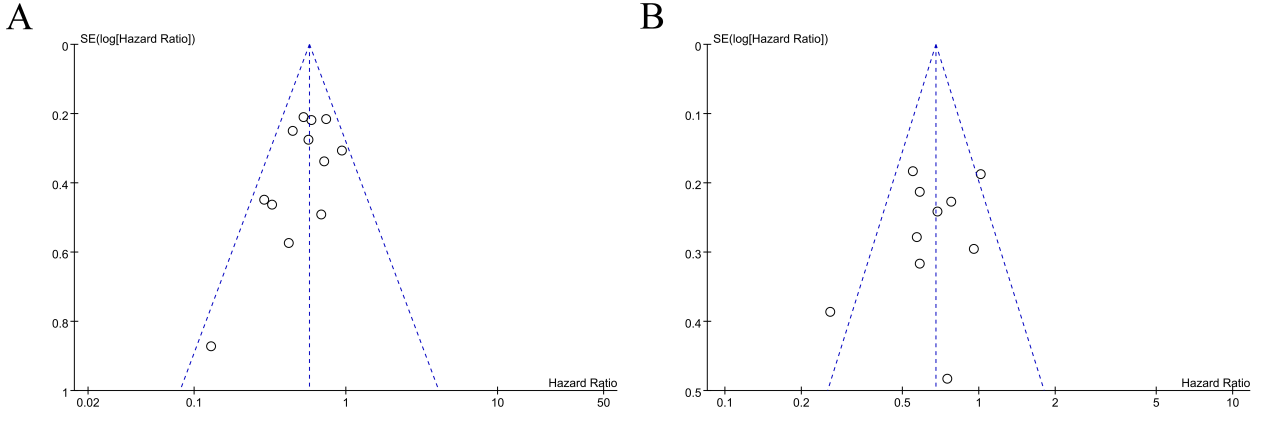
Supplementary Figure 1.** Funnel plots of ALC for OS(A) and PFS(B).

**
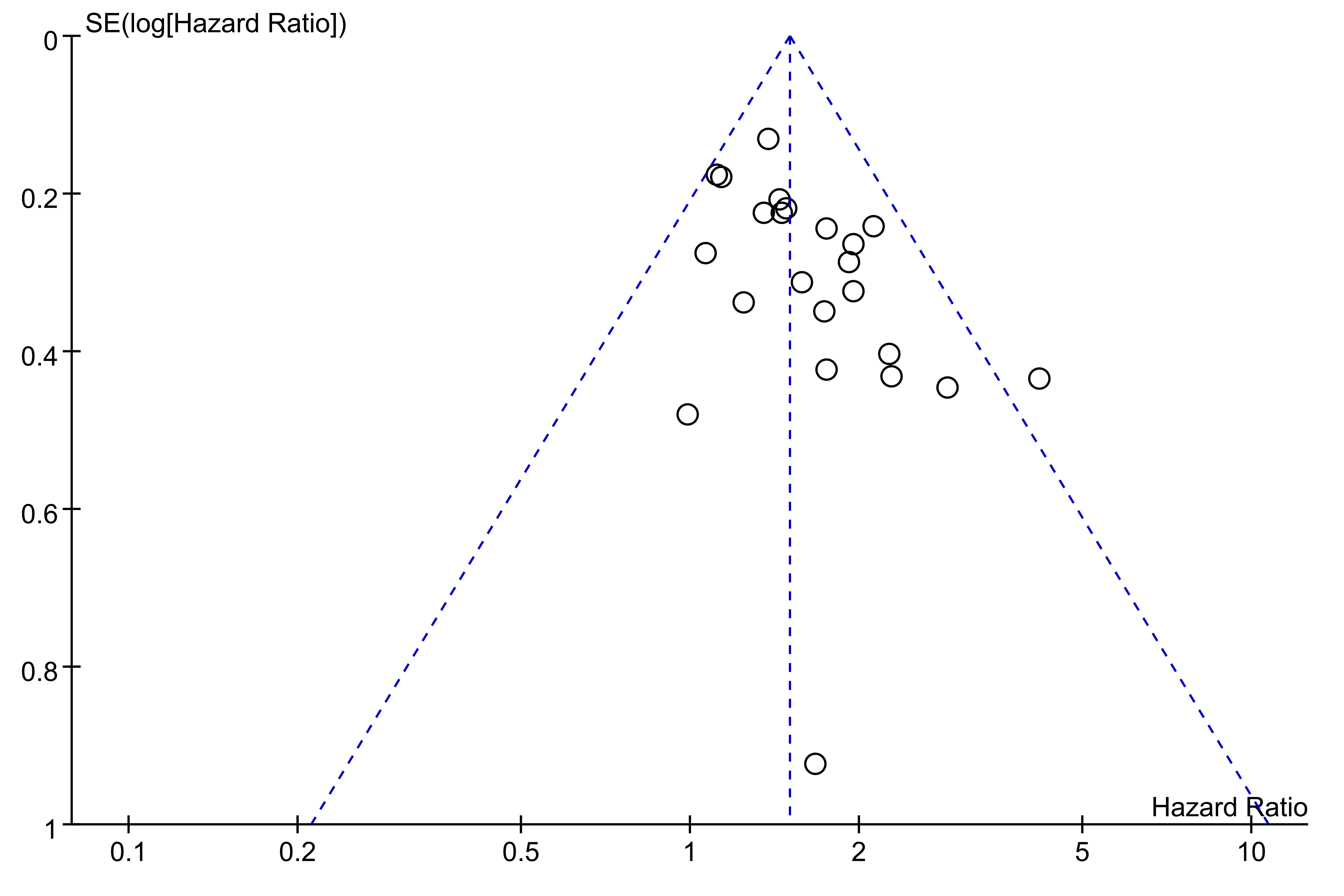
Supplementary Figure 2.** Funnel plots of NLR for OS.
